# Strategies for Community Education Prior to Clinical Trial Recruitment for a Cervical Cancer Screening Intervention in Uganda

**DOI:** 10.3389/fonc.2016.00090

**Published:** 2016-04-13

**Authors:** Sheona M. Mitchell, Heather N. Pedersen, Musa Sekikubo, Christine Biryabarema, Josaphat J. K. Byamugisha, David Mwesigwa, Malcolm Steinberg, Deborah M. Money, Gina S. Ogilvie

**Affiliations:** ^1^Department of Obstetrics and Gynecology, University of British Columbia, Vancouver, BC, Canada; ^2^School of Population and Public Health, University of British Columbia, Vancouver, BC, Canada; ^3^Department of Obstetrics and Gynecology, Mulago Hospital, Makerere University, Kampala, Uganda; ^4^Kisenyi Health Unit, Kampala, Uganda; ^5^Faculty of Health Sciences, Simon Fraser University, Burnaby, BC, Canada; ^6^Women’s Health Research Institute, Vancouver, BC, Canada

**Keywords:** cervical cancer screening, health education, qualitative research, participation, recruitment

## Abstract

**Introduction:**

Community engagement and education can improve acceptability and participation in clinical trials conducted in Kisenyi, Uganda. In preparation for a randomized controlled trial exploring different methods for cervical cancer screening, we explored optimal engagement strategies from the perspective of community members and health professionals.

**Methods:**

We conducted key informant interviews followed by serial community forums with purposeful sampling and compared the perspectives of women in Kisenyi (*N* = 26) to health-care workers (HCW) at the local and tertiary care center levels (*N* = 61) in a participatory, iterative process.

**Results:**

Key themes identified included format, content, language, message delivery, and target population. Women in Kisenyi see demonstration as a key part of an educational intervention and not solely a didactic session, whereas health professionals emphasized the biomedical content and natural history of cervical cancer. Using local language and lay leaders with locally accessible terminology was more of a priority for women in Kisenyi than clinicians. Simple language with a clear message was essential for both groups. Localization of language and reciprocal communication using demonstration between community members and HCW was a key theme.

**Conclusion:**

Although perceptions of the format are similar between women and HCW, the content, language, and messaging that should be incorporated in a health education strategy differ markedly. The call for lay leaders to participate in health promotion is a clear step toward transforming this cervical cancer screening project to be a fully participatory process. This is important in scaling up cervical cancer screening programs in Kisenyi and will be central in developing health education interventions for this purpose.

## Introduction

Cervical cancer remains the most common cancer among women in sub-Saharan Africa, which represents the majority of the global burden of this disease (1). Despite the availability in many countries of an effective primary prevention option, the HPV vaccine, and early secondary prevention interventions with screening, Uganda has one of the highest rates of cervical cancer in the world (2, 3). This is largely due to the competing health priorities and lack of health-care infrastructure to fully implement the national cervical cancer screening strategies. Although cervical cytology or traditional Pap smear screening has resulted in a huge reduction in cervical cancer in high-income countries, the infrastructure required in low- and middle-income countries (LMIC) remains a significant challenge (4). Cervical cancer screening is a rapidly evolving field, and currently cytology, visual inspection with acetic acid, and detection of high-risk strains of the human papillomavirus (HR-HPV) DNA are all screening tools recommended by the World Health Organization (WHO) (5). Consequently, program planners and decision makers are tasked with making context-specific decisions around which strategies to implement. Randomized controlled trials (RCTs) are largely accepted as the gold standard to inform evidence-based approaches for cervical cancer screening (6), and are increasingly being used to test public health policies and programs (7). The WHO has strongly endorsed a trial comparing self-collection for HR-HPV and VIA as a crucial next step to define optimal cervical cancer screening in LMIC (5, 6); however, implementation of large RCTs in LMIC provides significant challenges for researchers.

The *A*dvances in *S*creening and *P*revention of *Re*productive Cancers (ASPIRE) collaboration was created in 2006 as a community-based cervical cancer screening strategy using HPV self-collection and HPV-DNA testing as a feasible, acceptable, and cost-effective model to improve access to screening among women most at risk (8–11). Obstetrician gynecologists from Makerere University, working with researchers from the University of British Columbia (UBC), initiated partnerships with community leaders in Kisenyi, a densely populated urban community close to the center of Kampala, Uganda, known for significant poverty and health inequities. In 2013, the ASPIRE team began planning an RCT comparing self-collection-based HR-HPV testing to VIA in Kisenyi (12). In the development of an HPV self-screening program for women in this community, there was a voiced desire to create locally relevant and effective health education forums as a way to increase women’s knowledge and participation in the screening trial.

There is local evidence to suggest that health-care providers have low levels of knowledge regarding cervical cancer risk factors and screening (13). Previous ASPIRE studies have found that knowledge of HPV, cervical cancer, and perceived risk are low among women in the community of Kisenyi (8). This in turn could negatively impact the uptake and acceptability of screening interventions in the community. Conversely, by enhancing community education, barriers, such as embarrassment, associated with cervical cancer screening can be reduced in this setting (11). As such, it was deemed important to involve both health-care providers and community members in the design process for future health education around cervical cancer in this community. The objective of the study was to determine what would constitute an effective educational intervention for cervical cancer screening, increase participation in future screening programs, and optimize engagement in future trials.

## Materials and Methods

### Discussion Guides

**Figure 1:**
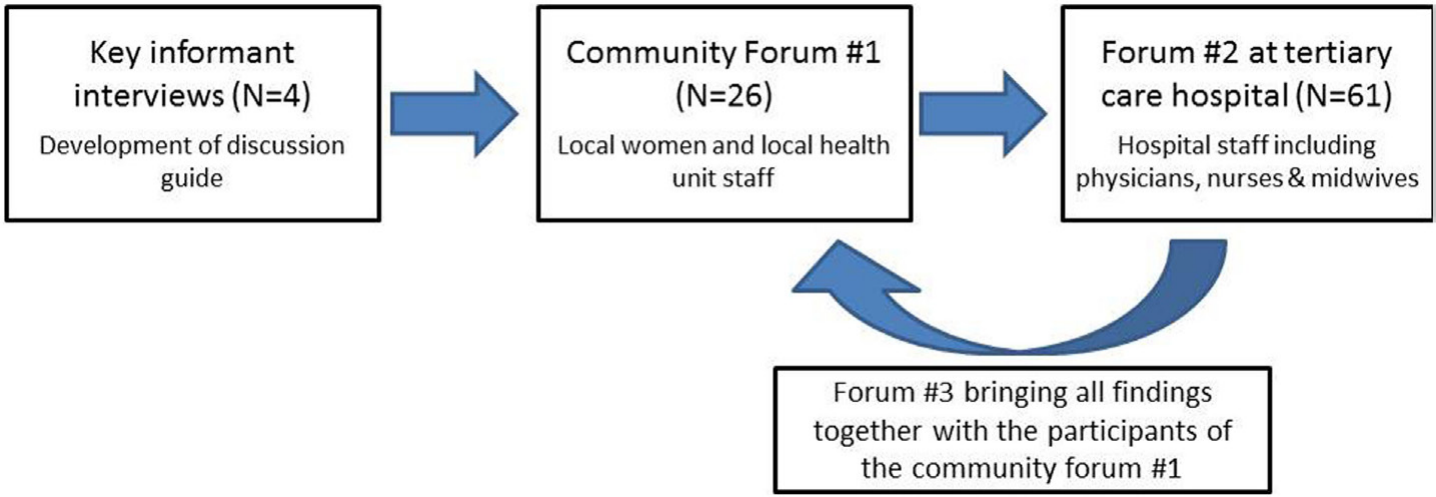
**Study flow diagram**.

The study consisted of a series of informant interviews and focus groups that were carried out in four stages: (1) key informant interviews to develop forum questions; (2) focus group with women from the community and staff at the local health unit; (3) focus group with health-care workers (HCW) at the tertiary care hospital (Mulago Hospital); and (4) debriefing session with women in the community (Figure 1). Key informant interviews with community health workers who had previously participated in the ASPIRE project, including a village chairperson and a member of Kampala city council (*N* = 4), were performed to develop discussion guides for the community forums (see Table 1). These were followed by three community forum discussions. Purposive sampling was used to select key community forum participants, most of whom had participated in the program’s screening activities in 2011.

**Table 1:** **Community forum discussion guide**.

(1) What is the ideal means/format for educating women on cervical cancer screening?
(2) What language should the education be delivered in?
(3) What content should the educational intervention include?
(4) Who should lead or implement the educational intervention?
(5) How should the educational messages be delivered?

Community forums were facilitated by UBC and Makerere University researchers with translation and cultural interpretation provided by a Ugandan project facilitator. All participants completed a brief demographic and consent form (see Table 2). The initial community forum held at the Kisenyi Health Unit (KHU) comprised local women and members of the clinical staff from the Health Unit (*N* = 26). The second forum held at Mulago Hospital, a tertiary referral center was entirely composed of health-care professionals (*N* = 61), including nurse midwives and obstetricians. The third forum was held at KHU and included the same participants as the first forum. It utilized themes identified from both of the initial two forums to present and to incorporate their reflections as part of an iterative process to ensure that the community members had full participation and the final say in what themes were gleaned from the forums.

**Table 2:** **Kisenyi community forum demographics (*N* = 27)**.

Median age (IQR) (years)	42.5 (35–49)
Education level	
No schooling or some primary school	4.76
Completed primary school	42.9
Completed secondary school	38.1
Post-secondary education	14.3
Previous involvement with ASPIRE (%)	90.9
Health-care workers (%)	45.4

The discussion guide used in the community forums contained open-ended questions that were guided by topics drawn from the key informant interviews on what aspects should be included in an effective educational intervention for cervical cancer screening and increase participation in future screening programs (Figure 1).

### Data Management and Analysis

All community forums were recorded and transcribed into NVivo 9 (QSR International Pty, Ltd.) qualitative software and coded. Since discussions were primarily carried out in Luganda, a Luganda speaker translated the discussions verbatim for transcription, which were reviewed by a second Luganda speaker. Organizational categories were developed prior to the community forums based on key informant interviews and included the topics of content, format, language, and message delivery. Thematic analysis was done of the transcribed forum data by initial sorting of nodes into substantive categories, which were descriptive in nature (14). Initial coding was performed by the Canadian researcher who performed transcription. Coding reliability checks were done after performing peer debriefing independently by both Ugandan and Canadian researchers. Ethics approval was obtained from the Institutional Review Boards of both UBC and Makerere University (H13-02117).

## Results

### Content

Community forum participants challenged the one-sided didactic communication methods commonly found in health education interventions, instead of emphasizing the importance of content that included demonstration. Specifically related to the method of screening being promoted by the ASPIRE project, which includes women collecting their own vaginal swabs for HPV testing, women wanted to have someone physically demonstrate with models how to perform the collection and be able to handle the swabs during the education intervention.

Demonstrating, asking ‘can this hurt me? How can I use it?’ … that is what everybody can understand. It doesn’t hurt so you can use it instead of explain it to them. – participant

Conversely, health-care providers at Mulago hospital put more emphasis on addressing perceived knowledge gaps with lay descriptions of the natural history of cervical cancer, including what age to start screening and treatment options at all stages of disease, including radiotherapy for more advanced stages. There was concern that women in Kisenyi did not have an accurate understanding of the anatomy of the reproductive system, and this was a priority focus for the education initiative to promote cervical cancer screening. There was also a desire to simplify and demystify cervical cancer screening described by a care provider at Mulago.

[As] health care workers we cannot assume that they will understand if we talk about uterus, now we talk about cervix, most of the women don’t appreciate this. – HCW

It’s extremely important to demystify the entire process of screening, putting it to mind that this is something that encroaches on the privacy of someone … we need to give information, very simple information on what exactly is involved in the screening process. – HCW

### Format and Message Delivery

Both women in Kisenyi and health-care workers mentioned a variety of formats for education interventions, including pamphlets, radio and television advertisements, use of local newspapers, and campaigns with loud speakers and announcers driving through Kisenyi, which are commonly used for other public health campaigns. Women in Kisenyi agreed that the most effective format would be a workshop or seminar that Kisenyi residents from different sub-villages could attend. Community participants quickly recognized the knowledge diffusion potential of these workshops or seminars.

For information, send some people – you only, you only (pointing to individuals) … so if there is a seminar, those people who have been in the seminar they will be the ones to go and tell other people. – Participant

Handouts, pamphlets, and other visual materials were described as important knowledge diffusion mechanisms to complement the seminars, and provide the opportunity for women who attended the workshop to share information with other men and women in the community that did not attend.

Facilitators of the proposed seminars differed between women in Kisenyi and health-care workers. The Mulago group suggested leadership by experts in the field who were aware of the local reality and had a clinical versus academic practice. Women in Kisenyi were much more eager to have facilitation by respected community members that are known to them. There was also a desire to have physicians share in facilitation who have been involved in the ASPIRE project for some time and have already built rapport and trust with participants in Kisenyi.

We should go through the chairpersons within the community so that for them it becomes easier to talk to people and mobilize people. And people believe in them. – Participant

### Language

Given the diverse population of Kisenyi with Somali women and migrants from other regions of Uganda where Luganda is not spoken, the issue of translation during the seminars and the importance of local terminology, regardless of language, were also discussed by the group in Kisenyi. The role of language was more of a priority for women in Kisenyi than for HCWs, as one woman in Kisenyi explained,

We use our local language [Luganda] so that they understand and Swahili I think, so we use three languages so everybody can understand. – Participant

Yes, to translate … even if someone uses English it can be explained locally so that everybody understands. – Participant

### Target Population

The community forum in Kisenyi agreed that there were many subpopulations in their community that could be targeted specifically, who may not be regular attenders at the KHU. The involvement of men in these educational interventions was a prominent theme in both groups in Kisenyi and HCW.

We can get those women from churches, mosques, through public places we can make cervical cancer awareness. We can ask her just to talk to her for ten minutes and talk about cancer of the cervix. – Participant

You make people know that women have other big problems concerning cancer, and it’s also good to let their husbands know what’s going on. When the husband knows about cancer of the cervix he can also send his wife to go for screening. – Participant

I’m wondering if the [community health workers] are going to [offer screening] to women at a time when the men are not at home … any of those who are involved sexually because if someone is walking into their house and the wife is doing that self-examination they could think of so many other things, given the culture we have, and it may be a detrimental factor to the whole project. So I think they must be in complement. – HCW

Another subpopulation, Somali women, was discussed in both focus groups as an important population to include with some specific challenges. The keys to overcoming these barriers include building rapport and connecting with key Somali individual who can act as cultural translators as described by a Kisenyi woman, and was supported by a Mulago physician.

I was involved in a government census, and they had to include Somali people and initially they did not want to participate but if you really spent time and talked to them carefully they would participate … If we explain to women that we are helping them to be tested for cancer and we take time to explain this to them when we enter into their houses then they will be willing to participate. – Participant

I’ve also worked a lot with Somali communities, one of the things that you can do to penetrate is use a woman. Use a peer Somali and you get as many as you can. Once they are comfortable with the project we have a few who can speak very good English who can be interpreters … once you get those they will tell many women. – HCW

## Discussion

### Format and Content

It remains unclear if the formats for a health education intervention discussed, such as workshops or seminars, are those that are the most effective or simply what women are familiar with and have experienced in the past. Many of the suggestions were similar between HCW and women in Kisenyi, which suggests that the format and content of previous education interventions may be their shared experience and what they are familiar with. Interestingly, the formats included both interactive education styles with demonstration and unidirectional message, for example, the use of public announcers. Variations in content between clinicians and community members are fairly intuitive with clinicians reflecting their training in physiology and the natural history of disease, whereas women were more interested in the logistics of how a self-collected swab is used and hearing from the personal experience of someone they respected. Incorporating aspects of both of these perspectives into a potential health education intervention is ideal. However, since community members are ultimately the knowledge users of education interventions, priority should be given to their ways of understanding to ensure that messages are delivered in a culturally appropriate fashion and taken up in a meaningful way. This holds the ultimate goal of moving toward community-driven health promotion from a predominantly provider driven in a transition to a truly participatory process.

### Demonstration

Demonstration of self-collection technique by lay leaders was also a key theme. This is supported by Bankole’s work in sub-Saharan populations that found participation in the demonstration of condom use had the strongest correlation with correct usage for HIV prevention (15). In the same vein, Lindemann has described that relying solely on the use of written instructions in the setting of condom use does not translate into appropriate use or increased knowledge (16). Women emphasized that interactive communication between community members and health-care workers that functioned reciprocally with information moving in both directions was the most powerful way to engage women in cervical cancer screening. It follows that when women feel empowered to step forward and interact with the HCW, their lived experience comes together with the clinical knowledge of the disease process and together these enable engagement with cervical cancer screening services. Involving lay leaders as integral members of the health education team is another step toward using primary care settings as opportunities for preventative health practice. Conversations with HCW can be an empowering and effective process to help community members translate health promotion messages into culturally appropriate language and messaging.

### Language

Language is intricately tied to cultural competence and communication. In our team’s previous experience in this community, the language around cervical cancer is already ambiguous, often referred to as “women’s cancer” without differentiation of cervix, uterus, or ovary (8). These linguistic challenges would be ideally clarified with both an expert in the field and a community member to find culturally appropriate yet medically accurate description to use in health education. The language of this communication was also a priority for women, with an emphasis on the localization of language and the creation of culturally relevant material. The importance of culture in improving the effectiveness of health communication is well known (17). Access to local terminology may not even be available to HCW who work at the KHU but ultimately requires community members whose lives are woven into the life of the community. With this type of two-way communication, there is much hope for engaging and retaining women in the screening program.

### Involvement of Male Partners

An example of the centrality of local knowledge in communication is the theme of including men in any educational sessions that are planned. Traditional women’s health educational events are solely targeted at women and fail to incorporate men in an attempt to improve discussion and foster openness among women who may be less likely to speak up in public settings when men are present (18). Themes from the community forums suggest that women in Kisenyi feel that the involvement of male partners is central to a successful cervical cancer screening program. HPV is intricately linked to sexual activity, and a partner’s reaction or knowledge of HPV and especially their response to a woman self-collecting a vaginal swab could determine whether or not a woman engages with screening.

### Community Engagement in Clinical Trials Research

There are ethical issues at play when conducting research in developing countries, which demand our attention. At the core of these issues, according to Emanuel et al., are standard of care that should be used in developing countries; access and availability of proven interventions; and the quality of informed consent (19). The responsibility of researchers in this setting goes beyond the trial and study participants but rather extends to the community in which studies are being conducted (20). There are a multitude of factors that increase the risk of exploiting participants and communities through multinational research in developing countries, which include lack of access to health services, poverty, and lack of knowledge and education (19). It is important that research institutions work in a collaborative manner to reduce the risk of exploitation by ensuring that research is acceptable and reflects health needs of the community. Enhancing community participation through locally appropriate education is only one way that ASPIRE aims to maintain high ethical standards. Others include shared operational decision making between Canadian and Ugandan investigators, locally informed research priorities, scalable interventions, and government partnerships to work toward uptake beyond the research period. The results of this study were used to enhance ASPIRE community education program prior to successfully recruiting 500 women for a RCT in this community (12).

### Limitations

A limitation of this study was that the women who participated in the Kisenyi-based community forum were those who had previously participated in the ASPIRE project and are a highly engaged population. As a result, their views may reflect more of a familiarity with health education interventions as they have previously participated in a variety of engagement and education workshops. Although these women are a highly engaged population, there are still barriers to a truly participatory process as there is an inherent imbalance between them and the Canadian and Ugandan researchers facilitating the discussion. Additional limitations are the loss of richness and detail in the Luganda discussion during the translation process to English for data analysis. Attempts were made to limit this by having two separate Luganda speakers review the final transcriptions.

## Conclusion

It is well documented that there is a lack of cervical cancer education, particularly in LMIC, and that this lack of knowledge can be a barrier to uptake in communities (21). Health education interventions conducted in Nigeria resulted in improved knowledge and attitudes about cervical cancer (22). In this study, lack of awareness was cited as the main reason for never having accessed screening, and the number of participants with a good knowledge of cervical cancer rose from 2 to 70.5% after education. Similarly, ASPIRE has noted that community education and engagement has been a key catalyst for acceptability and uptake of interventions (8, 9). Despite this, few studies address strategies that can be used to strengthen communication and education in these settings, emphasizing the importance of the current study findings.

This study was completed with the goal of developing locally relevant and effective health education interventions around cervical cancer, and we have found variation in perspectives of women in Kisenyi compared to the health workers. ASPIRE was able to integrate the findings from the community forums into a new community engagement strategy, which is proving to be successful in priming the community for the introduction of cervical cancer screening, educating women about this important health issue, and engaging community leaders to become champions for the ASPIRE project. In recent qualitative work done by our group describing the role of embarrassment as a barrier to screening uptake, community members cited ongoing education and engagement as a key intervention to decrease this barrier and subsequently increase screening uptake (11). Applications of this specific study are currently being implemented to the ongoing screening activities currently being rolled out in Kisenyi and will inform future community education forums prior to recruitment for clinical trials at other sites. All future trials will incorporate study findings with evaluations of pre-study engagement and education in a variety of populations.

## Author Contributions

All the authors have made a substantial contribution to (a) the conception and design and/or the analysis and interpretation of data, (b) drafting the article or revising it critically for intellectual content, and (c) approved the version submitted.

## Conflict of Interest Statement

The authors declare that the research was conducted in the absence of any commercial or financial relationships that could be construed as a potential conflict of interest.

## References

[1] World Health Organization. The Global Burden of Disease: 2004 Update. (2009). Available from: www.who.int/evidence/bod

[2] Uganda Ministry of Health. Strategic Plan for Cervical Cancer Prevention and Control in Uganda: 2010-2014. (2010). Available from: http://www.rho.org/files/PATH_Uganda_cxca_strat_plan_2010-2014.pdf

[3] WHO/ICO. Human Papillomavirus and Related Cancers in Uganda. Summary Report 2010 [Online]. (2010). Available from: http://www.hpvcentre.net/statistics/reports/UGA.pdf

[4] Sankaranarayanan R Ferlay J. Worldwide burden of gynaecological cancer: the size of the problem. Best Pract Res Clin Obstet Gynaecol (2006) 20:207–25. 10.1016/j.bpobgyn.2005.10.007 16359925

[5] World Health Organization. Comprehensive Cervical Cancer Control: A Guide to Essential Practice. 2nd ed (2014). Available from: http://apps.who.int/iris/bitstream/10665/144785/1/9789241548953_eng.pdf?ua=1 25642554

[6] World Health Organization. WHO Guidelines for Screening and Treatment of Precancerous Lesions for Cervical Cancer Prevention. (2013). Available from: http://apps.who.int/iris/bitstream/10665/94830/1/9789241548694_eng.pdf?ua=1 24716265

[7] Tollefson J Revolt of the randomistas. Nature (2015) 524:150–3. 10.1038/524150a 26268176

[8] Mitchell S Ogilvie G Steinberg M Sekikubo M Biryabarema C Money D. Assessing women’s willingness to collect their own cervical samples for HPV testing as part of the ASPIRE cervical cancer screening project in Uganda. Int J Gynaecol Obstet (2011) 114:111–5. 10.1016/j.ijgo.2011.01.028 21669428

[9] Ogilvie GS Mitchell S Sekikubo M Biryabarema C Byamugisha J Jeronimo J Results of a community-based cervical cancer screening pilot project using human papillomavirus self-sampling in Kampala, Uganda. Int J Gynaecol Obstet (2013) 122:118–23. 10.1016/j.ijgo.2013.03.019 23731506

[10] Mitchell SM Sekikubo M Biryabarema C Byamugisha JJ Steinberg M Jeronimo J Factors associated with high-risk HPV positivity in a low-resource setting in sub-Saharan Africa. Am J Obstet Gynecol (2014) 210:81.e1–7. 10.1016/j.ajog.2013.08.038 23999419

[11] Teng FF Mitchell SM Sekikubo M Biryabarema C Byamugisha JK Steinberg M Understanding the role of embarrassment in gynaecological screening: a qualitative study from the ASPIRE cervical cancer screening project in Uganda. BMJ Open (2014) 4:e004783. 10.1136/bmjopen-2014-004783 24727360 PMC3987737

[12] Moses E Pedersen H Mitchell S Sekikubo M Mwesigwa D Singer J Uptake of community-based, self-collected HPV testing vs. visual inspection with acetic acid for cervical cancer screening in Kampala, Uganda: preliminary results of a randomised controlled trial. Trop Med Int Health (2015) 20:1355–67. 10.1111/tmi.12549 26031572

[13] Mutyaba T Mmiro F Weiderpass E. Knowledge, attitudes and practices on cervical cancer screening among the medical workers of Mulago Hospital, Uganda. BMC Med Educ (2006) 2006(6):13. 10.1186/1472-6920-6-13 16509979 PMC1413529

[14] Maxwell J Qualitative Research Design: An Interactive Approach. eBook. Thousand Oaks: SAGE (2012).

[15] Bankole A Ahmed F Neema S Ouedraogo C Konyani S. Knowledge of correct condom use and consistency of use among adolescents in four countries in Sub-Saharan Africa. Afr J Reprod Health (2007) 11:197–220. 10.2307/25549730 18458741 PMC2367135

[16] Lindemann D Harbke C Are written instructions enough? Efficacy of male condom packaging leaflets among college students. Health Educ J (2012) 72:180–8. 10.1177/0017896912437300

[17] Kreuter M McClure S. The role of culture in health communication. Annu Rev Public Health (2004) 25:439–55. 10.1146/annurev.publhealth.25.101802.123000 15015929

[18] Nguyen T Mcphee S Bui-Tong N Luong T Ha-Iaconis T Nguyen T Community-based participatory research increases cervical cancer screening among Vietnamese-Americans. J Health Care Poor Underserved (2006) 17:31–54. 10.1353/hpu.2006.0091 16809874

[19] Emanuel EJ Wendler D Killen J Grady C What makes clinical research in developing countries ethical? The benchmarks of ethical research. J Infect Dis (2004) 189:930–7. 10.1086/381709 14976611

[20] Dal-Re R Ndebele P Higgs E Sewankambo N Wendler D Protections for clinical trials in low and middle income countries need strengthening not weakening. BMJ (2014) 349:g4254 10.1136/bmj.g4254 24996885 PMC4688422

[21] Lim JN Ojo AA. Barriers to utilisation of cervical cancer screening in Sub Sahara Africa: a systematic review. Eur J Cancer Care (Engl) (2016). 10.1111/ecc.12444 26853214

[22] Abiodun OA Olu-Abiodun OO Sotunsa JO Oluwole FA. Impact of health education intervention on knowledge and perception of cervical cancer and cervical screening uptake among adult women in rural communities in Nigeria. BMC Public Health (2014) 14:814. 10.1186/1471-2458-14-814 25103189 PMC4133628

